# PREVALENCE OF SUICIDAL IDEATION IN PATIENTS WITH SCHIZOPHRENIA TREATED AT THE PSYCHIATRIC OUTPATIENT CLINIC OF A TEACHING HOSPITAL

**DOI:** 10.1192/j.eurpsy.2023.2236

**Published:** 2023-07-19

**Authors:** F. A. Borghi, L. C. Signorini, L. Z. Alves, R. C. C. Oliveira, G. M. Araujo Filho

**Affiliations:** 1 Psychosis Outpatient Clinic, Base Hospital; 2 FAMERP - Medical School of São José do Rio Preto, São José do Rio Preto - São Paulo, Brazil

## Abstract

**Introduction:**

Patients with schizophrenia tend to have high rates of suicidal ideation (SI), which consists of thoughts of self-destruction, which increase the risk of self-extermination.

**Objectives:**

To determine the prevalence of SI and investigate associated factors in a sample of patients with schizophrenia.

**Methods:**

Descriptive and cross-sectional study, in which 49 patients with the condition were selected by convenience, treated at the Psychosis Outpatient Clinic of the Base Hospital of São José do Rio Preto/SP, between August/2021 and March/2022. The following were applied: 1) Sociodemographic Questionnaire, 2) Suicide Ideation Section of the Columbia Suicide Risk Assessment Scale (SISC-SSRS), 3) Suicide Risk Questionnaire from the Mini International Neuropsychiatric Interview (SRQ-MINI). Data were analyzed quantitatively (descriptive statistics and non-parametric tests; p<0.05). The study was approved by the local Research Ethics Committee.

**Results:**

The age of the participants ranged from 17 to 72 years (mean=45.8 ±14.02), most were male (n=34;69.4%), had not completed elementary school (n=25; 51%), did not have a paid job (n=41; 83.7%) and had a family income of up to three minimum wages (n=23;46.9%). 40.8% (n=20) reported at least one suicide attempt. According to the SISC-SSRS, in the last month: 22.9% (n=11) wished they were dead; 18.8% (n=9) thought about killing themselves; 12.5% (n=6) considered how they could perform the act; 10.4% (n=5) had intention and active planning; and 10.4 (n=5) persisted for the purpose of execution. The mean of affirmative answers was equal to 0.75 (±1.55). In turn, in the SRQ-MINI, 79.6% (n=39) had a score indicating low risk for suicide, 18.4% (n=9) high risk and 2% (n=1) moderate risk. The overall mean was 5.77 (±10.31), which indicates a moderate risk for suicide. There was a non-significant negative correlation between the risk of suicide and the factors of education (r= -0.20; p=0.15) and family income (r= -0.21; p=0.13). There was a significant positive correlation (r=0.81; p=0.0001) between the SISC-SSRS and SRQ-MINI, which indicates that despite the adapted use of the instrument, there is consistency and reliability in the results.

**Conclusions:**

The sample showed low rates of active SI and variation between low and moderate risk for suicide. SI should be asked to patients with schizophrenia, with a view to preventing suicidal behavior.

**Disclosure of Interest:**

None Declared

